# Hypotonic, Acidic Oxidizing Solution Containing Hypochlorous Acid (HClO) as a Potential Treatment of Hailey-Hailey Disease

**DOI:** 10.3390/molecules24244427

**Published:** 2019-12-04

**Authors:** Samantha Cialfi, Salvatore Calabro, Matteo Franchitto, Azzurra Zonfrilli, Isabella Screpanti, Claudio Talora

**Affiliations:** 1 Department of Molecular Medicine, Sapienza University of Rome, 00161 Rome, Italymatteo.franchitto@uniroma1.it (M.F.); azzurra.zonfrilli@uniroma1.it (A.Z.); isabella.screpanti@uniroma1.it (I.S.); 2 Center of Life Nano Science Sapienza, Istituto Italiano di Tecnologia, 00161 Rome, Italy

**Keywords:** Hailey–Hailey disease, oxidative-stress, keratinocytes

## Abstract

Hailey–Hailey disease (HHD) is a rare, chronic and recurrent blistering disorder, characterized by erosions occurring primarily in intertriginous regions and histologically by suprabasal acantholysis. Mutation of the Golgi Ca^2+^-ATPase *ATP2C1* has been identified as having a causative role in Hailey–Hailey disease. HHD-derived keratinocytes have increased oxidative-stress that is associated with impaired proliferation and differentiation. Additionally, HHD is characterized by skin lesions that do not heal and by recurrent skin infections, indicating that HHD keratinocytes might not respond well to challenges such as wounding or infection. Hypochlorous acid has been demonstrated in vitro and in vivo to possess properties that rescue both oxidative stress and altered wound repair process. Thus, we investigated the potential effects of a stabilized form of hypochlorous acid (APR-TD012) in an in vitro model of HHD. We found that treatment of ATP2C1-defective keratinocytes with APR-TD012 contributed to upregulation of Nrf2 (nuclear factor (erythroid-derived 2)-like 2). Additionally, APR TD012-treatment restored the defective proliferative capability of siATP2C1-treated keratinocytes. We also found that the APR-TD012 treatment might support wound healing process, due to its ability to modulate the expression of wound healing associated cytokines. These observations suggested that the APR-TD012 might be a potential therapeutic agent for HHD-lesions.

## 1. Introduction

Hailey–Hailey disease (HHD, OMIM 16960), also indicated as benign familial pemphigus, is an autosomal dominantly inherited dermatosis manifesting in the 3rd to 4th decades of life [1]. The overall incidence and prevalence of HHD is unknown although some authors have reported an incidence between 1:40,000 and 1:50,000 [2,3]. HHD is a characterized by red scaly areas that can be painful and itchy and can lead to superficial blisters and eroded areas of the skin. This disease often has a remission and recurrence pattern, which may be constant in some patients. HHD is associated with the loss of a single copy of *ATP2C1*, a gene that is likely essential in humans, as more severe phenotypes are found in patients who suffer clonal loss of both copies of the gene. Consistently, mice embryos homozygous for null mutations in ATP2C1 die with defects in neural tube closure, while heterozygotes show susceptibility to squamous cell tumors, a phenotype observed rarely in humans with Hailey–Hailey [4,5]. The gene, located on the long arm of chromosome 3, 3q21-q24 region, encodes the human secretory pathway Ca^2+^/Mn^2+^ ATPase, hSPCA1 [6]. Although ATP2C1 is mostly localized to the Golgi apparatus, it regulates also endoplasmic reticulum (ER) Ca^2+^ stores with effects on both Golgi and ER functions. In keratinocytes the lack of ATP2C1 leads to the loss of cell-to-cell adhesion (acantholysis) among the cells of the suprabasal layer of epidermis probably due to a retraction of keratin intermediate filaments from the desmosomal plaques [1]. Although ATP2C1 mutations are 100% penetrant, currently there is no treatment known to be effective in reducing the cutaneous manifestations of HHD. The standard of care (SOC) treatment consists in either topical or oral administration of corticosteroids often used in combination with topical/systemic antimicrobial agents. External factors, such as sweating, UV exposure, friction and superinfection with bacteria, fungi and viruses play an important role in exacerbations and persistence of lesions [2,7,8]. Calcium regulates the proliferation and differentiation of keratinocytes both in vivo and in vitro, thus it is not surprising that ATP2C1 mutations affect the skin. However, it is unclear how ATP2C1 loss selectively affects keratinocyte homeostasis. Oxidative stress represents a hallmark of the keratinocytes derived from the lesions of HHD patients and it could be associated to the decreased action of some detoxifying systems [9,10,11,12,13]. Particularly, in the lesional-derived keratinocytes of HHD patients, the expression of detoxifying genes is down-regulated [9,10,11,12,13]. As oxidative stress is thought to play a pivotal role in promoting the skin lesions of Hailey–Hailey, counteracting oxidative-stress could be a viable therapeutic approach for HHD. HHD lesional-derived keratinocytes in addition to being characterized by increased oxidative stress also show decreased expression levels of both NOTCH1 and NRF2 [9,10,11,12,13]. However, in vitro, the inhibition of ATP2C1 expression in HaCaT cells resulted in increased levels of activated NOTCH1 and decreased expression of NRF2, indicating that NOTCH1 downregulation in HHD-lesions could represent a secondary event that might be required at a later stage of the lesion development [10]. Both NOTCH1 and NRF2 factors are important determinant of skin homeostasis; NOTCH signaling is an essential regulator of keratinocyte growth and differentiation and NRF2 activation regulates antioxidant genes transcription directly. Skin lesions that do not heal and by recurrent skin infections are main pathological feature of HHD keratinocytes, indicating that they may be not able to counteract insults such as wounding or infection. After injury an increase of local cytokine production from keratinocytes occurs [14,15,16]. In skin wounds in order to regulate the re-epithelization process, crucial cytokines as interleukin (IL)-6 and transforming growth factor (TGF)-beta, are produced locally [17,18,19,20,21,22,23]. It has been shown that HHD lesion-derived keratinocytes were defective in wound-induced cytokine production [10]. Therefore, these data indicate that keratinocytes derived from the HHD lesions are defective in managing both oxidative-stress response and wound signal that ultimately could contribute to the poor healing of HHD lesions. NRF2 is widely regarded to be an oxidative stress-activated transcription factor and its essential role is to keep a physiological redox homeostasis. Transient and moderate oxidative stress may up-regulate genes involved in antioxidant and cytoprotective pathways through the activation of the transcription factor Nrf2 [24]. Hypochlorous acid (HClO) in addition to a strong antimicrobial activity is an oxidant generated in the pathogenesis of many disorders [25,26]. After exposure of cells to HClO, NRF2-mediated antioxidant response is activated and resulted in increased protein levels of NRF2, as well as in an increase in the expression of its target genes [25,26,27]. In addition HClO has both pro-inflammatory and anti-inflammatory properties [25,26,27]. APR TD012 is a water-based solution obtained by an electrolysis process (Tehclo Technology). This method allows us to obtain the active compound HClO deliverable in a hypotonic, acidic (pH: 2.5–3.0) and oxidizing (Oxidation Reduction Potential: 1000–1300 mV) solution. Due to the great relevance of both oxidative stress and altered pattern of cytokines expression in HHD-derived keratinocytes, we evaluated if the APR TD012 treatment was able to recover the oxidative-stress as well as the altered pattern of cytokine expression of ATP2C1-defective keratinocytes. With this aim, we analyzed the response of ATP2C1-defective keratinocytes to the addition of this hypotonic acidic oxidizing solution containing HClO. We investigated the effect of APR TD012 treatment on proliferation rate, NRF2 expression, antioxidative-stress activity and cytokines expression of ATP2C1-defective keratinocytes. We found that APR TD012 solution was able to restore NRF2 defective expression, differentially affected the expression of TGFbeta1 and TGFbeta2 and had a favorable effect on ATP2C1-defective keratinocyte proliferation and on in vitro wound assay. These features, together with the well-known antimicrobial activity of HClO, identify APR TD012 as a potential agent in the treatment of HHD-lesions.

## 2. Results

### 2.1. Levels of Oxidative Stress in ATP2C1 Defective Keratinocytes Treated with APR TD012

**Figure 1:**
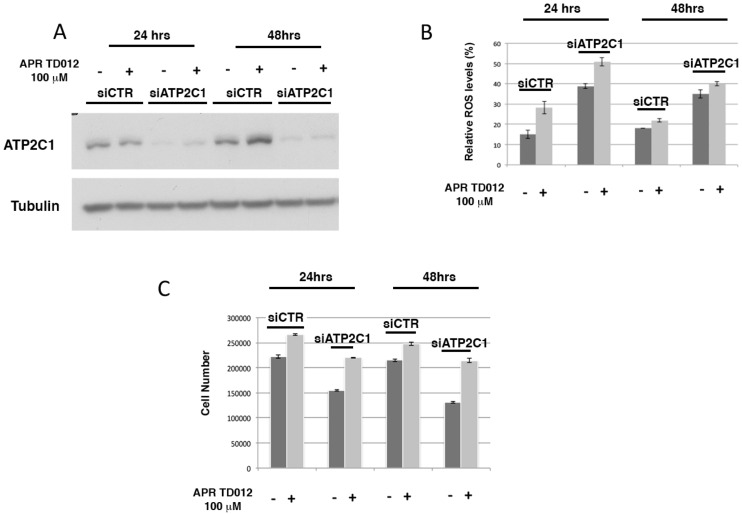
ROS and proliferative rate after APR TD012 treatment. (**A**) Immunoblot analysis of ATP2C1, in siCTR and siATP2C1 HaCaT cells. Tubulin expression was used as control for equal loading. (**B**) Cells were analyzed by Fluorescence Activated cell sorting (FACS) and % of ROS levels is shown. (**C**) Cell number of siCTR and siATP2C1 keratinocytes was analyzed by Trypan-blue assay. The averages ± standard error of two independent experiments in triplicate are shown.

To test the effect of hypotonic acidic oxidizing solution containing HClO (APR TD012) we used small interfering RNA (siRNA)-ATP2C1-treated keratinocytes as an in vitro model of Hailey–Hailey disease. Towards this aim, HaCaT cells were transfected with siCTR and siATP2C1 and then treated with APR TD012. As previously observed, we found that ATP2C1 loss increased oxidative stress (Figure 1A,B). After 24 h of transfection, the percentage of 2’, 7’-dichlorofluorescin diacetate (DFCA)-positive cells in siATP2C1 cells reached ≅ 38%, whereas only ≅ 15% of the siRNA-CTR control cells were DFCA-positive. In this model analysis of Reactive Oxygen Species (ROS) levels, APR TD012 treatment brought an increased oxidative-stress in both siCTR (≅28%) and siATP2C1 (≅52%) treated HaCaT cells (Figure 1B). At the 48 h time point of transfection, the percentage of DFCA-positive cells in both siCTR and siATP2C1 cells were similar to ROS levels observed at 24 h (Figure 1B), whereas the levels declined in both siCTR and siATP2C1 cells when APR TD012 was present in the medium (Figure 1B). Interestingly, we reported that HHD lesion-derived keratinocytes were hypo-proliferative compared to the non-lesion-derived keratinocytes [10]. HClO and hypotonic stress has been shown to have a positive effect on keratinocyte migration and proliferation [25], and both these events are defective in Hailey–Hailey disease [10]. Therefore, we tested whether APR TD012 treatment could influence ATP2C1-defective keratinocytes proliferation. We confirmed that siATP2C1 cells had reduced proliferation compared to siCTR treated cells (Figure 1C). Interestingly the treatment of siATP2C1 cells with APR TD012 rescued the defective proliferation of siATP2C1-treated HaCaT cells (Figure 1C).

### 2.2. Effects of APR TD012 on the NRF2/Antioxidant Defense Pathway

**Figure 2:**
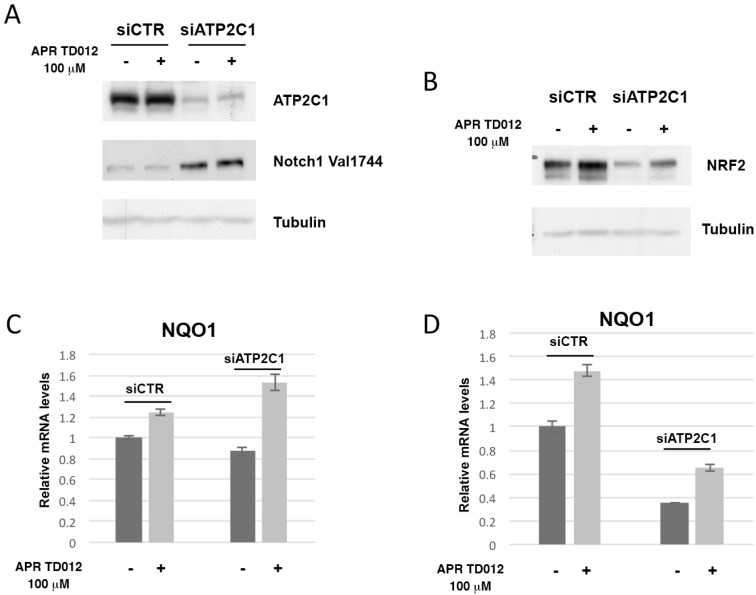
Effects of APR TD012 on NRF2 and its target expressions. Immunoblot analysis of ATP2C1, Notch1 Val1744 (**A**) and NRF2 (**B**) in siCTR and siATP2C1 HaCaT cells. Tubulin expression was used as control for equal loading. qRT-PCR analysis of NQO1 mRNA expression levels in siCTR and siATP2C1 HaCaT (**C**) or NHEK (**D**) cells. The values are expressed as fold changes of siATP2C1 cells vs. the siCTR. The averages ± standard error of two independent experiments are shown.

Previously we have observed that NOTCH1 expression levels were increased in siATP2C1 cells [10]. Additionally, the expression of NRF2 was decreased in siATP2C1 treated keratinocytes [10]. These events could play an important role in HHD development since ATP2C1 loss would trigger a mechanism that results in NOTCH1 activation and DNA damage response inhibition. As a result of NFR2 down-modulation increased ROS levels produced DNA damage up to a threshold that keratinocytes could not repair, which would then promote lesion manifestation. Thus, we investigated how a treatment with hypotonic acidic oxidizing solution containing HClO might affect both NOTCH1 and NRF2 expression. We found that siATP2C1 treatment of HaCaT keratinocytes showed an increased expression of active NOTCH1 when compared to control cells (Figure 2A). However, there were no significant differences between vehicle and APR TD012 treated siATP2C1-cells. Conversely, the NRF2 protein expression levels were significantly higher after treatment with APR TD012 (Figure 2B). In conjunction with NRF2 increased expression, we observed that APR TD012 treatment induced the expression of NRF2 target gene NQO1 in both HaCaT and primary human keratinocytes (Figure 2C,D). These data suggest that APR TD012 promoted an antioxidant defense response by activating the NRF2 pathway although this protective circuitry failed to counteract the high ROS levels observed in ATP2C1-defective keratinocytes.

### 2.3. Effects of APR TD012 on the Expression of Keratinocyte-Derived Cytokines

**Figure 3:**
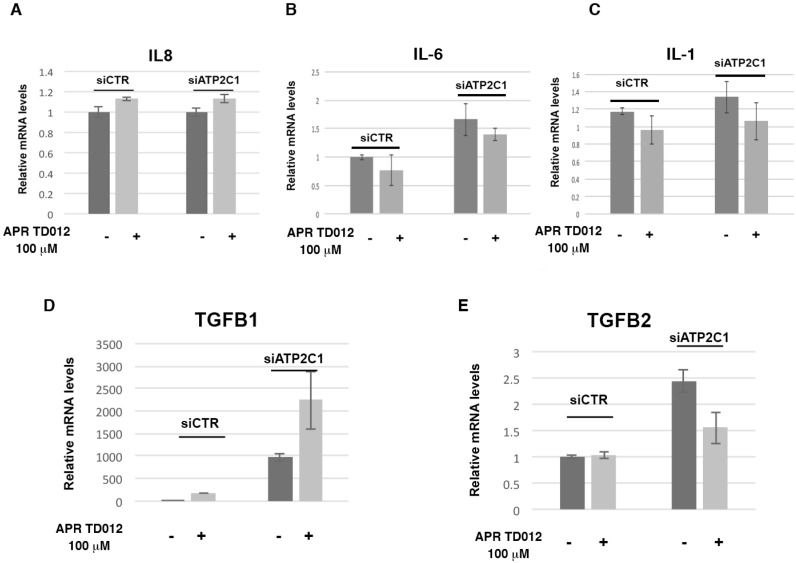
Effects of APR TD012 solution on proinflammatory cytokine in ATP2C1 defective keratinocytes. (**A**) IL-8, (**B**) IL-6, (**C**) IL-1 (**D**) TGFβ-1 and (**E**) TGFβ2 were quantified by q-RT-PCR assay. The values are expressed as fold changes of siATP2C1 cells vs. the siCTR. Data are expressed as mean ± SD of two independent experiments performed in triplicate.

The success of the wound healing process depends on growth factors, cytokines and chemokines. HHD lesions are characterized by deregulated cytokine expression and decreased repair properties [10]. Thus, we investigated the influence of APR TD012 treatment on the pattern of deregulated cytokines on ATP2C1-defective keratinocytes. We have shown an altered expression levels of the pro-inflammatory cytokines IL-1, IL-6, IL-8, TGFβ1 and TGFβ2 in ATP2C1 defective keratinocytes [10]. However, no significant differences in IL-1, IL-6 and IL-8 levels were observed between vehicle and APR TD012 treated siATP2C1-cells (Figure 3A–C); Conversely, in the siATP2C1 cells the mRNA levels of TGFβ1 and TGFβ2 were significantly higher than those of the control siCTR-control cells (Figure 3D,E). Interestingly, a significant difference in TGFβ1 and TGFβ2 levels were observed between the vehicle and APR TD012 treated cells. In particular, the upregulation of TGFβ1 expression was observed in the siATP2C1 cells compared to the siCTR cells and, interestingly, even more after APR TD012 treatment (Figure 3D).

Likewise, the levels of the TGFβ2 cytokine were significantly higher in the siATP2C1 treated cells than those of the control cells (Figure 3E). On the contrary, while the TGFβ2 expression remained unaffected in siCTR cells, a decreased expression levels were observed between the vehicle treated and APR TD012 treated siATP2C1-cells (Figure 3E). These data suggest that APR TD012 might influence the pattern of proinflammatory cytokines expression in HHD-keratinocytes.

### 2.4. In Vitro Wound Healing Potential of APR TD012 on ATP2C1-Defective Keratinocytes

**Figure 4:**
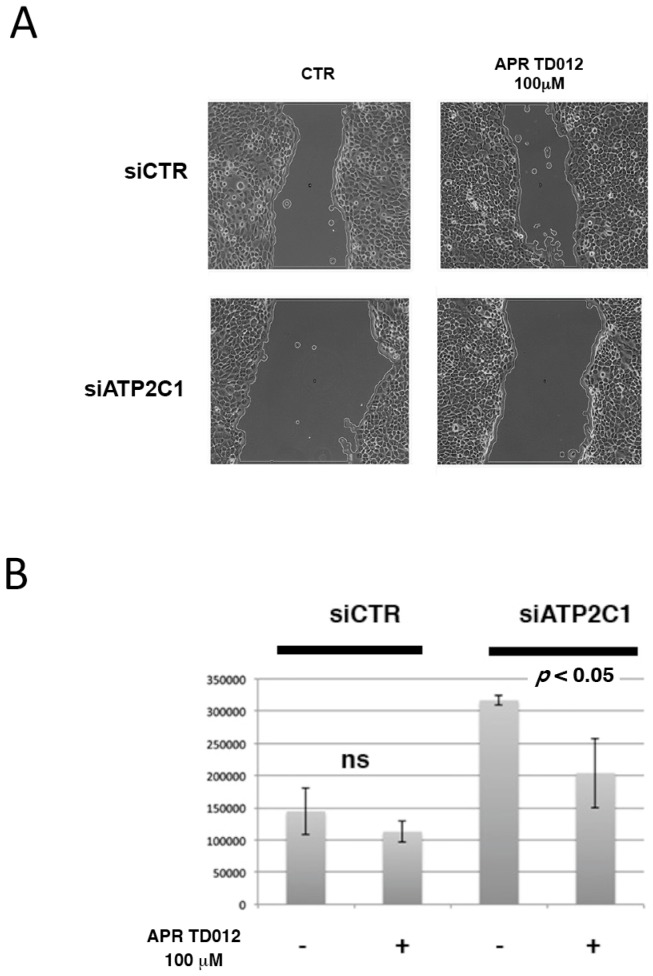
In vitro scratch assay (×40 magnification). (**A**) Representative images of siCTR and siATP2C1 cell migration into the scratched area after treatment with vehicle or APR TD012. (**B**) Quantitative analysis of the migration rate was analyzed with the use of ImageJ software. Data are expressed as mean ± standard deviation from three individual experiments.

HHD keratinocytes are characterized by impaired healing repair [10] and migration of cells is critically involved in wound repair; thus we conducted a scratch assay to analyze the healing process of siATP2C1-treated keratinocytes in the presence of APR TD012. After 24 h exposure to the APR TD012 solution, we observed that in the control siCTR-treated cells, the rate of migration was not influenced by the treatment (Figure 4). The values given were calculated based on the scratch coverage after 24 h. The migration analysis showed that for the siATP2C1 cells the vehicle-treated cells showed a significant decrease in migration with the value of 50%, when compared with the siCTR-treated cells. When the APR TD1012 treated cells were compared, they showed a significant increase in migration with the migration rate of 34% when compared to siATP2C1 vehicle-treated cells, also if there was still difference when compared with siCTR-control cells.

The results showed that the proliferation and migration of siATP2C1 treated keratinocytes were significantly higher in APRTD012 cells than the control group. These data suggested that APR TD012 enhanced ATP2C1 defective keratinocyte proliferation and migration. 

## 3. Discussion

HClO is thought to promote wound-healing process and to both promote oxidative-stress and consequently the activation of the NRF2/antioxidant pathway [15,25,26,27]. We have found that both these events are defective in Hailey–Hailey disease [10]. Our previously published results had implicated oxidative stress and the response to it as contributing factors to the presentation of HHD. It is known that transient and moderate oxidative stress may up-regulate genes involved in antioxidant and cytoprotective pathway through the activation of the transcription factor Nrf2 [24]. We hypothesized that APR TD012, a hypotonic acidic oxidizing solution containing HClO might be an effective treatment for HHD by restoring the activation of the NRF2 pathway. Additionally, previous observations suggested that HClO improves wound healing process since is highly active against bacterial, viral and fungal human pathogens [15,25,26,27]. HHD is characterized by skin lesions that do not heal and by recurrent skin infections, indicating that HHD keratinocytes might not respond well to challenges such as wounding or infection. Thus in this study, we aimed to assess the potential use of APR TD012 in patients with HHD. To test this hypothesis, we first analyzed whether there was evidence of an effect of hypotonic acidic oxidizing solution containing HClO treatment in an in vitro model of HHD. We first investigated the effects of APR TD012 treatment on the oxidative-stress levels of ATP2C1 defective-keratinocytes. We found that oxidative-stress increased in APR TD012 treated keratinocytes with both functional and defective ATP2C1. It has been reported that the oxidative action of HClO induces the activation of the NRF2/antioxidant defense pathway thereby, paradoxically, reducing oxidative-stress [15,25,26,27]. Thus, to determine a possible functional link between APR TD012 and NRF2 and the presence of oxidative stress in HHD, we performed a series of experiments in HaCaT keratinocytes transfected with either siCTR or siATP2C1 and then treated with either vehicle or APR TD012. As previously shown, we found that NRF2 was downregulated in keratinocytes with interfered *ATP2C1* gene. Interestingly, when siATP2C1 transfected keratinocytes were treated with APR TD012, NRF2 expression was restored. We found that after treatment, the proliferation of ATP2C1 defective keratinocytes resembled that of control keratinocytes. Together, these results indicate that APR TD012 solution can act directly on keratinocytes to protect them from HHD defects, consistent with previous observation suggesting that increased NRF2-pathway increases the defense mechanism of HHD-keratinocytes [9,10].

An important finding of our study was the observation that APR TD012 influenced the expression of both TGF-β isoforms β1 and β2. Although TGF-β isoforms signal through the same cell surface receptors, they display distinct functions during wound healing in vivo through mechanisms that have not been fully elucidated [28,29]. Numerous studies have highlighted the role of TGF-beta signal in cutaneous wound healing [28,29]. The well-characterized role of TGF-β1 and -β2 on promoting wound healing has provided the basis for the use of TGF-β1 or -β2 as potential therapeutic [28,29]. Interestingly, we found that in ATP2C1 defective cells, APR TD012 treatment decreased TGF-β2 expression while increased TGF-β1 expression. Although it is likely more complex than this, since TGF-β isoforms display distinct functions during wound healing, it is thought that the ratio of TGF-β isoforms will differently influence the wound healing process [30]. Thus, also if we did not address this aspect our results indicate that APR TD012 treatment altering the ratio of TGF-β isoforms could positively affect the resolution of HHD lesions. It has been found that loss of TGFβ-2 signaling in keratinocytes led to an accelerated re-epithelialization of full thickness excisional wounds accompanied by an increased proliferation in keratinocytes at the wound edge [31]. Furthermore, impaired TGFβ signaling in keratinocytes reduces apoptosis in re-epithelialized wounds of transgenic animals [31]. A speculative prospective is represented by the possibility that the ability of APR TD012 to decrease TGF-β2 while increasing TGF-β1 expression could be a means to restore the proliferative potential of ATP2C1 defective keratinocytes improving the wound process. To support this hypothesis, we performed a scratch wound healing assay after APR TD012 treatment. The results showed a reduction of width of wound in siATP2C1 cells demonstrating an improved proliferation through this pharmacological approach. The wound healing requires elimination of microrganisms, removing damaged cells and tissue and restoring the skin barrier, three needed steps to restore tissue integrity and APR TD012 might help to carry out these complex of processes.

Together, these results provided a rationale to test the use of APR-TD012 solution for the treatment of HHD lesions.

## 4. Materials and Methods

### 4.1. Cell Culture

HaCaT keratinocyte-derived cell line were cultured in DMEM medium supplemented with 10% fetal bovine serum (FBS), 5% L-Glutamine, 2% penicillin and streptomycin, at 37 °C with 5% CO^2^.

### 4.2. Cell Culture and Transfection

HaCaT cells (70–80% confluent) were transfected using the Lipofectamine RNAiMAX transfection Reagent according to the manufacturer’s instructions (Thermo Fisher Scientific, Waltham, MA, USA) using 100 nmol L^−1^ small interfering RNAs (siRNAs) for validated human ATP2C1 (L-006119-00; Thermo Scientific/Dharmacon, Lafayette, CO, USA) and corresponding control scrambled siRNAs. Cells were analyzed after 48 h of transfection for ROS detection or Western blot as indicated. In the time 24 and 48 h point experiment HaCaT cells (20–30% confluent) were incubated 6 h with the Lipofectamine RNAiMAX transfection reagent according to manufacturer’s instructions (Thermo Fisher Scientific, MA USA). Then cells were untreated or treated with APR TD012 solution for either 24 or 48 h and analyzed for ROS detection or Western blot as indicated.

### 4.3. Cell Treatment with APR TD012

APR TD012 (batch 2147) was diluted 1:10 in order to reach the concentration of 100 μM in the assays.

### 4.4. Cell Viability Assay

HaCaT cells (siCTR and siATP2C1) were grown and used for cell viabilities assay at the second passage by using Trypan-blue based assay. After 24 h of transfection with siATP2C1 or siCTR, cells were treated 24 h with 100 μM APR TD012. As control samples, cells were treated with equal volumes of the vehicle (H2O). Trypan-blue assays were performed in technical triplicates and figures show the averages ± SEM of at least two biological replicates.

### 4.5. Measurement of ROS Accumulation

Intracellular production of ROS was measured using cell-permeable fluorescent dyes, 5-(and-6)-chloromethyl-2’, 7’dichlorodihydrofluorescein diacetate, acetyl ester (CMH2DCFDA Molecular Probes). When this dye is oxidized by ROS in cells, their fluorescent signals increase. For the assay, after transfection and addiction of APR TD012, HaCaT cells were treated with CMH2DCFDA for 30 min, in the dark at 37 °C. Next, cells were washed twice with PBS, trypsinized and fluorescence was measured using flow cytometry (excitation at 488 nm, emission at 515–545 nm). Data analysis was performed with CellQuestPro software (BD Biosciences, Milan, Italy), and the mean fluorescence intensity was used to quantify the responses. A minimum of 10,000 cells were acquired for each sample, excluding the dead population.

### 4.6. Western Blot Assay

Cells were lysed in Tris HCl 20 mM pH 7.5, NaCl 150 mM, EDTA 1 mM pH 8, Triton 1%, NaF 30 mM, Na3VO4 1 mM, PMFS 1mM and protease inhibitors (Merck life Science, Milan Italy); samples were centrifuged at 13,000 rpm for 15 min and supernatant was collected. Quantification was performed with Bradford assay (Bio-Rad). Lysates were denatured at 95 °C and separated through SDS-PAGE on 8% acrylamide gel. After transfer to a polyvinylidene difluoride (PVDF) membrane, proteins were immunoblotted using standard procedures. The primary antibodies for ATP2C1 and NRF2 were purchased from [10] Abcam, Cambridge, UK; Notch1Val1744and Tubulin were purchased from Cell Signaling Technology, Beverly, MA, USA and Sigma Aldrich, Milan, Italy respectively.

### 4.7. RNA Analysis and Reverse Transcriptase-Polymerase Chain Reaction

Total RNA was isolated from cells, in guanidine isothiocyanate (Trizol reagent, Thermo Fisher Scientific, MA USA) and further processed by reverse transcriptase polymerase chain reaction (RT-PCR) as described [32]. Each sample was analyzed in triplicated by qRT-PCR and in at least two independent experiments. qRT-PCR was performed at the opportune annealing temperature with the primers indicated below, with SensiFAST SyBr Hi-ROX kit (Bioline, UK) or with specific TaqMan MGB primers/probe using Taqman gene expression assay (Thermo Fisher Scientific, MA USA). hIL-1 and hIL-6 primers were previously described in [10].

### 4.8. Statistical Analysis

Each experiment was repeated at least two times independently. All results were expressed as means SD, and *p* < 0.05 was used for significance. One-Way ANOVA analysis for independent samples was used to determine statistical significance.

### 4.9. Primers

hTGFB1 qPCR Fw:CAGAAATACAGCAACAATTCC;hTGFB1 qPCR Rev:CTGAAGCAATAGTTGGTGTC;hIL8 qPCR Fw:AAGGAAAACTGGGTGCAGAG;hIL8 qPCR Rev:ATTGCATCTGGCAACCCTAC;hGAPDH qPCR Fw:TGCACCACCAACTGCTTAG;hGAPDH qPCR Rev:GAGGCAGGGATGATGTTC;TGFbeta2: Hs00234244_m1.

## 5. Conclusions

Hailey-Hailey disease (HHD) is a Rare disease and currently there is no treatment known to be effective in reducing the cutaneous manifestations of the disease. We have gathered compelling evidence indicating that oxidative-stress plays a pivotal role in promoting the skin lesions of Hailey–Hailey disease. HHD-keratinocytes show decreased expression levels of NRF2 and NRF2-regulated antioxidant enzymes leading to the accumulation of ROS. The standard of care (SOC) treatment consists in either topical or oral administration of corticosteroids often used in combination with topical/systemic antimicrobial agents, but without reversing the pathological process. In this line, the discovery of new therapies aimed to target the pathogenic mechanism underlying the disease is undoubtedly an important goal, in order to provide better and more efficient treatment conditions for HHD patients. In this context, we investigated the potential effects of a stabilized form of hypochlorous acid (APR-TD012) in an in vitro model of HHD. We found that treatment of ATP2C1-defective keratinocytes with APR-TD012 contributed to up-regulation of Nrf2. Additionally, APR TD012-treatment restored multiple defects observed in siATP2C1-treated keratinocytes. These observations suggested that the APR-TD012 might be a potential therapeutic agent for HHD-lesions.
